# A summary guide for detecting and reducing nitrous oxide infrastructure leaks in healthcare facilities

**DOI:** 10.1177/0310057X261449257

**Published:** 2026-06-14

**Authors:** Eugenie A. Kayak, Rebecca E. McIntyre, Forbes McGain, Justin J. Skowno, Steven J. Gaff, Gemma Cooper, Madeleine Skellern, Arthur Wyns

**Affiliations:** 1 Department of Critical Care, University of Melbourne, Melbourne, Australia; 2 Department of Anaesthesia and Pain Management, Royal Children’s Hospital, Melbourne, Australia; 3 Department of Anaesthesia and Intensive Care, Western Health, Melbourne, Australia; 4 Department of Anaesthesia, The Children’s Hospital at Westmead, Sydney, Australia; 5 School of Child and Adolescent Health, Faculty of Medicine and Health, University of Sydney, Sydney, Australia; 6 Department of Anaesthesiology and Perioperative Medicine, Alfred Health, Melbourne, Australia; 7 National Heath, Sustainability and Climate Unit, Australian Centre for Disease Control, Australian Government, Canberra, Australia; 8 School of Public Health, University of Sydney, Sydney, Australia; 9 Melbourne School of Population and Global Health, University of Melbourne, Melbourne, Australia

**Keywords:** Nitrous oxide, inhaled anaesthetics, greenhouse gases, environmental impact, sustainability

## Abstract

Leaks from nitrous oxide (N_2_O) infrastructure have been identified as a significant contributor to the greenhouse gas emissions of healthcare facilities. Recent studies in Australia and in the United Kingdom have found at least half (and often more than 70%) of the N_2_O supplied to many healthcare facilities leaks from medical pipelines and associated infrastructure before clinical administration. To assist in addressing this issue, the University of Melbourne, in collaboration with the Interim Australian Centre for Disease Control and the Australian Government’s Department of Health and Aged Care, published *Detecting and reducing nitrous oxide leaks in healthcare facilities – a practical guide* (the ‘Guide’) in 2024. This article summarises the Guide and best practice approaches for detection and reduction of N_2_O leaks in healthcare facilities across Australia. It is intended to assist clinicians and facility managers to identify appropriate methods to test for N_2_O leaks, make an informed choice about the most appropriate way to supply N_2_O to a facility and reduce waste from N_2_O leaks in their healthcare facility.

## Introduction

Nitrous oxide (N_2_O), a gas used for labour analgesia, sedation and dentistry, and as an adjunct in general anaesthesia, is a significant contributor to health system greenhouse gas emissions, accounting for around 20% of the direct (scope 1) emissions of the Australian health system.^1^ In Australian healthcare facilities N_2_O is often supplied from a central store of cylinders and delivered through a network of rigid pipelines to areas of a facility for administration. Pipeline supply can also be present in non-clinical areas of healthcare facilities for legacy reasons as facilities are renovated or upgraded.

Leaks in N_2_O piping infrastructure have been identified as a significant contributor to the greenhouse gas emission footprint of anaesthetic gas use in healthcare and are financially wasteful. Recent studies in Australia and in the United Kingdom have found at least half (and often more than 70%) of the N_2_O supplied to healthcare facilities leaks from infrastructure before clinical administration.^2–9^ Given that N_2_O is both colourless and odourless, leaks need to be actively detected.

International interest in addressing N_2_O leaks gained momentum in 2021, when an audit of 16 hospitals in the Lothian National Health Service revealed that waste via leaks accounted for over 95% of N_2_O procured.^4,10^ Further audits at 38 sites in Scotland demonstrated that 83–100% of purchased N_2_O was lost via infrastructure leaks.^4,5^ Numerous healthcare facilities in Scotland have subsequently decided to decommission their piped supply of N_2_O.^4^ There is growing international recognition of the imperative to reduce waste from N_2_O leaks and an increasing body of opinion, most recently in a joint statement published by the Australian and New Zealand College of Anaesthetists, the Australian Society of Anaesthetists and the New Zealand Society of Anaesthetists, that piped N_2_O does not have a place in a sustainable high-quality health system.^11–15^

The Australian Government committed to reducing emissions from medical N_2_O in the 2023 National Health and Climate Strategy, which sets out a whole-of-government plan for addressing the health and wellbeing impacts of climate change, whilst also mitigating the contribution of the health system to climate change through the generation of greenhouse gas emissions in care delivery.^1^ Action 4.13 of the Strategy commits to working to improve patient care, protect health care staff and reduce greenhouse gas emissions from N_2_O, by:

(a) Reducing waste from N_2_O leaks;
(b) Dealing with venting, the practice of releasing unused N_2_O when cylinders are returned for refill;
(c) Educating on appropriate use.

## Background methodology

As part of the work to implement the National Health and Climate Strategy, in 2024 the Australian Government Department of Health and Aged Care worked with the University of Melbourne to publish a practical guide to detecting and reducing N_2_O leaks in healthcare facilities.^16^ This article summarises key recommendations from the Guide as well as recent Australian progress and cases studies.

## Australian Standards

Australian Standard 2896:2021 (AS 2896:2021) sets out procedures for installation and testing of non-flammable medical gas pipeline systems.^17^ AS 2896:2021 informs the Australasian Health Facility Guidelines, which act as an overarching guide describing all required elements and relevant standards that should be adhered to in the construction and maintenance of healthcare facilities.

Under AS 2896:2021, monitoring and maintenance of medical gas pipeline systems is limited to visual checks, pressure gauge checks and the ‘soapy water bubble test’ (4.13.5). The last, which involves spraying detergent onto N_2_O pipes and valves to search for leaks identified as bubbles, provides only a rudimentary indication of the presence of leaks in specific locations and does not test the system in its entirety or allow for the size of any given leak (i.e. the volume of N_2_O being leaked) to be accurately determined.

Australian healthcare facilities that have passed the AS 2896:2021 maintenance standards have subsequently been shown to have major leaks when applying alternative testing methods for N_2_O leaks.^6–8^ This might reflect that the Australian Standards currently do not require regular ongoing leak testing of the entirety of the rigid pipeline network after commissioning (except for a requirement to pressure test after major modifications or prolonged downtime). It might also reflect that the Australian Standards currently do not provide specific guidance on the use of more accurate and comprehensive leak testing methodologies than the ‘soapy water bubble test’.^17^

The additional methods described in the Guide and this article can significantly improve the sensitivity by which N_2_O leaks are detected as part of an ongoing maintenance regime.

In recognition of the new understanding of N_2_O leakage from medical pipelines, the Australasian Health Facility Guidelines (AHFGs) were updated in 2024. The guidelines now state that reticulated N_2_O and associated scavenge outlets are not mandatory for a healthcare service, and that point-of-care cylinders can meet clinical requirements for the majority of healthcare facilities. In addition, the new AHFGs require that consideration be given to both measurement and monitoring of N_2_O usage, and the management of N_2_O leakage.^18^

## Potential sources of leaks

Leaks can occur along the N_2_O infrastructure of healthcare facilities (Table 1) with increasing facility age and inadequate maintenance schedules likely to increase the risk of leaks. While leaks from the manifold-pipeline system (the central pipe fitting which connects multiple gas cylinders to their points of use throughout the healthcare facility) appear to be more common, leaks can occur at other locations, including at wall outlets or at the point of clinical administration, such as N_2_O cylinders left open when attached to an anaesthetic machine.

**Table 1.** Likely sites of nitrous oxide (N_2_O) leaks within healthcare facilities.

Likely sites of N_2_O leaks	Likely sources of leakage at site
Manifold pipeline system	At the connection between individual gas cylinders and the manifold pipeline system.
O-Rings	At wall outlets, in pendants (such as the Non-Interchangeable Screw Thread O-rings) or in flexible tubing (usually a blue flexible tube running from an outlet to the anaesthetic machine).
Operating theatre pendants	At the connection to the pipework or outlet, often hidden behind a ceiling or wall.
Isolating valves in the pipeline system	Leaks between pipelines and isolating valves.
Wall outlets	There might be unused wall outlets in areas originally used for patient care that have since been converted to other uses without removing the pipework supply.
Therapeutic equipment attached to gas pipelines	Leaks within the equipment.
Damaged or obsolete pipeline	In sections of the pipeline that connect to outlets, have isolating valves or connect to a manifold.

## Detecting N_2_O leaks in a healthcare facility

The following initial steps are recommended in advance of attempting to detect leaks, to obtain an overview of the existing N_2_O infrastructure and history of supply:

1. Engage with the healthcare facility engineering department and/or facility manager to obtain a servicing schedule for the N_2_O pipeline and an up-to-date pipeline infrastructure map to help identify each outlet.
2. Clarify whether the healthcare facility engineering department does the servicing or whether they outsource this work to an external contractor. If external, confirm that the contracted servicing agency adequately tests for leaks, and identify the testing method(s) being used.
3. Obtain N_2_O purchasing data from the healthcare facility or directly from the supply company with as much detail as possible, such as cylinder size and weight and volume of N_2_O.

While it is necessary to have purchasing data only for the Discrepancy Method (described below), displaying purchasing data graphically by amount of N_2_O purchased over time (e.g. per month or year) can help identify the likelihood of leaks from changes in purchasing patterns (adjusting for any trends or cycles in clinical administration).

When detecting N_2_O leaks in healthcare facilities, it is essential to take a multidisciplinary approach by engaging a wide range of staff members throughout the process, including representatives from engineering and infrastructure, bioengineering, pharmacy, administration, procurement, anaesthesia, midwifery and obstetrics, paediatrics, emergency services, clinical governance, sustainability, safety and quality and others.

Four methods to detect N_2_O pipeline leaks have been described in the literature. These methods are summarised below and in Table 2 with further detailed descriptions found in the 2024 Guide.^16^

**Table 2.** Methods to detect nitrous oxide (N_2_O) pipeline leaks.

Method	Description	Practical considerations	Key benefits	Key limitations
Method 1: Discrepancy	Calculates discrepancy between volume of N_2_O purchased and volume (estimated or measured) of clinical use.	• Must include all areas of clinical use. • N_2_O use ideally obtained from ‘Super User’ menu of anaesthetic machines or electronic medical record. • If clinical use cannot be directly measured, estimates can be used based on published data (e.g. 500l per labour, 60l per paediatric sedation in Emergency Department).	Quick and user-friendly way to estimate total volume of leak.	• Does not identify physical site(s) of leaks • Does not include residual N_2_O in returned cylinders. • Might rely on imprecise estimations.
Method 2: Cylinder Weighing	Reduction in cylinder weight is tracked and compared with administered volumes for the same period.	Requires an industrial scale, for example, a 150kg scale, with a sensitivity of 10g to weigh a single G sized N_2_O cylinder attached to the manifold.	• Accurate. • Provides data on total volume of leak. • Accounts for residual N_2_O in returned cylinders.	• Ideally requires a period of no clinical use. • Does not identify physical site(s) of leaks. • Requires engineering assistance and specialised equipment.
Method 3: Pressure Testing	Pressure change in a fixed volume of pipework is tested during a period of no clinical use.	Measurement of pipeline pressure changes/decreases over 4 h (similar to the testing that occurs as part of pipeline commissioning).	Can test sub-sections of the pipeline (e.g. one operating theatre) or entire system.	• Requires at least a 4 h period of no clinical use. • N_2_O alarms might be activated during testing. • Requires involvement of clinical staff and infrastructure managers.
Method 4: Flow Monitoring	Flow meters are installed into the system to directly measure flow.	• Flow meters can be installed at the main manifold to facilitate detection of whole system leak or elsewhere to measure for leaks from other isolated parts of the system. • Flow meters can also be used at the point of administration to measure clinical use.	• Can assist in locating leaks (depending on location of meter). • Allows for real-time continuous measurement. • Mobile equipment can be shared by multiple facilities.	• Requires installation of purpose-built flow meters. • Associated cost and time might be limiting.

### Discrepancy Method

The Discrepancy Method was developed by the Nitrous Oxide Project in the United Kingdom.^4^ It calculates the difference between purchased amounts and clinically administered amounts of N_2_O, with discrepancy indicating a leak. It is a relatively quick and user-friendly method to indicate whether a leak is present.

Clinical administration amounts may be obtained by interrogating anaesthesia workstations. If this is not possible then estimations of clinical use, whilst likely less precise, have been used in the literature.^19^ Discrepancies greater than 15% might indicate the existence of a leak (or multiple leaks) in the N_2_O delivery infrastructure.

Other limitations of this method are that the resulting leak estimates do not take into account residual N_2_O in manifold cylinders when they are returned to the gas suppliers, and that physical site(s) of leaks are not identifiable without a subsequent leak site detection process.

**Table:** 

**Case study: Footscray Hospital^7^**
In 2021, Footscray Hospital in Melbourne found about 75% of total purchased hospital N_2_O was lost to leaks. Data was obtained from the ‘Super User’ menu screen of anaesthetic machines. The emissions associated with this N_2_O leakage were equivalent to over 75,000kg of carbon dioxide per year. An external engineering contractor was engaged and sprayed detergent onto the N_2_O pipes and all delivery systems, searching for leaks identified as bubbles. One leak was immediately identified near the main manifold and subsequently addressed.
Fixing the single N_2_O leak resulted in a greater reduction in greenhouse gas emissions than many other suggestions for reducing emissions elsewhere in the hospital, such as converting from sevoflurane to total intravenous anaesthesia, and required minimal financial investment. This experience highlights the strong value proposition for the detection of N_2_O leaks in healthcare facilities.

### Cylinder Weighing Method

The Cylinder Weighing Method was developed at the Alfred Hospital, Melbourne, in 2022.^8^ It detects N_2_O leaks via two similar methods: (a) weighing a manifold cylinder supplying N_2_O to determine depletion during a period when no N_2_O is administered, for example, overnight; or (b) weighing a manifold cylinder over a set time period when N_2_O is being administered and calculating the discrepancy between measured (via weight) cylinder depletion and N_2_O administration data.

This method relies on the fact that a regulator at the manifold maintains a constant pressure in the N_2_O pipeline network, hence any leak will lead to cylinder depletion and reduction in weight. An industrial scale, for example a 150 kg scale with a sensitivity of 10 g, is required to weigh a single N_2_O cylinder attached to the manifold.

Like the Discrepancy Method, this method provides information on leaks across a facility rather than identifying the physical site(s) of leaks. However, unlike the Discrepancy Method it does not include residual N_2_O contained in cylinders in the leak estimates.

This method is ideally undertaken during a period of no N_2_O clinical administration, which might be difficult in healthcare settings where continuous access to N_2_O supply is required.

### Pressure Testing Method

The Pressure Testing Method detects N_2_O leaks by measuring the pressure decrease in a fixed volume system during a period of no clinical administration, at constant temperature. During the test, the N_2_O supply is disconnected or isolated from the gas pipeline. Close consultation with clinical staff and infrastructure managers is required to plan and implement this testing method, as there is the potential for activation of low pressure N_2_O alarms. This method might need to be conducted out of hours, during a period of no clinical use for piped N_2_O, which might not be possible in healthcare settings where continuous access to N_2_O supply is required.

This method is based on a protocol developed in 2023 by the Green Theatres Project at the Fiona Stanley and Fremantle Hospitals in Western Australia. The full protocol is available on the website of the Green Theatres Project.^20^

Using this method, individual departments or areas can be checked for leaks (e.g. checking one operating theatre at a time), or the entire N_2_O delivery system can be checked as a system.

The suggested testing time is at least 4 h for each location being examined. An anaesthetic machine with a digital readout of N_2_O pipeline pressure can be used to measure the system pressure during the test.

### Flow Monitoring Method

The Flow Monitoring Method utilises purpose-built flow meters to assist in detecting both N_2_O leaks and/or the amount of N_2_O that is clinically administered (where anaesthetic machine records are not available). This method was developed by Wong and coauthors at Sunshine Hospital, Melbourne and subsequently adapted and implemented at the Sydney Children’s Hospital Network and the Royal Women’s Hospital, Melbourne.^19,21^

The flow monitoring method can be used to measure flow at any point, from the supply of nitrous oxide at the manifold, to a clinical delivery device connected to a wall outlet at the point of care. Coriolis flow meters are used to directly measure N_2_O flow rates, with high accuracy, either for the entire facility or for isolated areas.^22^

By installing a purpose-built flow meter at the point of the N_2_O supply infrastructure (e.g. at the central cylinder manifold) N_2_O supply can be measured, which might reflect supply more accurately than procurement data.

Clinical administration of N_2_O can also be measured by installing a purpose-built flow meter at the point of N_2_O clinical administration (i.e. wall outlets). The use of a threaded stainless steel sleeve enables the leakproof attachment of the N_2_O flow meter to N_2_O piping. Data obtained are used to accurately assess supply and clinical administration, and leaks can then be calculated using the Discrepancy Method.

The Flow Monitoring Method can be used to provide real-time, continuous measurement and, depending on the location of the flow meter, can also be used to locate leaks. It also has the potential to provide high-precision leak estimates (depending on the sensitivity of the flow meter). Given that the equipment is mobile, a single flow meter can be used across multiple facilities. Alternatively, permanent installation of leak detection equipment can be considered, to assist subsequent identification of leaks in real time.

**Table:** 

**Case study: The Children’s Hospital at Westmead, Sydney^21^**
A N_2_O flow meter was installed at the Children’s Hospital at Westmead in early February 2024, producing high resolution (1 min) and high fidelity (10–100 ml/min) data on N_2_O use for the entire hospital over a period of several months. The average N_2_O flow measured between 03:00 h and 04:00 h was used as a daily reference for a period of no clinical administration. Flow data are regularly cross-checked with purchasing data and usage data from anaesthetic machines.
The detection of continuous flow outside of periods of high N_2_O use indicated the presence of N_2_O leaks. The flow meter measurements also demonstrated a steady reduction in clinical N_2_O usage over time, which might have been caused by ongoing education and changes in clinical practice.

## Recommended next steps

Once a healthcare facility has identified the presence of a leak, using one of the four methods described, it is recommended to undertake a cost–benefit analysis to determine the best approach for a facility to reduce waste from N_2_O leakage. This could include:

- Complete decommissioning of existing N_2_O pipelines;
- Minimising N_2_O pipelines to supply only areas with a high-volume need (e.g. birthing suites);
- Use of portable cylinders to supply N_2_O at the point of care where possible;
- Avoiding installation of new N_2_O pipelines;
- Avoiding the use of N_2_O where there is no impact on patient care.

As part of any cost–benefit analysis, consideration should be given to how to incorporate the environmental cost of greenhouse gas emissions into the analysis as well as the cost of routine testing and servicing. These costs should be communicated clearly to all staff who procure, store and use N_2_O. The preferences of health practitioners and user groups most likely to require continued use of N_2_O—such as maternity staff and patients—should also be considered before implementing any major changes to N_2_O infrastructure.

In considering next steps, it is important to collaborate closely with engineering staff, facility management teams, and anaesthetic and other departments that administer N_2_O, to ensure clear roles and responsibilities for testing and reporting. Regular testing (every 3–6 months) will help detect new N_2_O leaks, allow for alternative leak detection methods to be tried and compared if needed and enable a better understanding of N_2_O use over time. Consultation and collaboration with the wider health workforce will also be important, including identifying cases of unnecessary or low-value use of N_2_O and education on appropriate use.

If zones in the healthcare facility are identified where no (or negligible amounts of) N_2_O is administered, consider whether the isolation valve for that zone could be switched off, effectively isolating these areas from the rest of the N_2_O infrastructure.

Healthcare organisations should also consider an organisational policy that outlines the recommended uses of N_2_O administration across their facilities. This could include appropriate indicators and a governance framework for monitoring and overseeing N_2_O use. To further drive changes in N_2_O administration patterns, the organisational policy would ideally encompass training of health practitioners, and implementation of sustainable procurement policies and reporting frameworks. Combining a whole-of-organisation approach of this kind with appropriate change communication and health workforce training would support tangible and sustained reductions in N_2_O administration while maintaining the quality and safety of care delivery.

## Decommissioning pipelines

Where N_2_O supply is required, healthcare facilities should consider moving away from piped N_2_O and instead supplying N_2_O via cylinders at the point of clinical administration. This would enable decommissioning of the entire N_2_O pipeline infrastructure and the associated financial and environmental benefits.

Hospitals in the United States and the United Kingdom that have decommissioned their N_2_O pipelines and moved to point-of-care cylinders have achieved large reductions in their N_2_O purchasing and greenhouse gas emissions as a result.^4,23^

Before decommissioning N_2_O pipeline infrastructure, facilities should consider:

- Existing anaesthetic machines might not have a yoke for N_2_O cylinders to connect to. In these cases a point-of-care cylinder on a mobile cart, fitted with a regulator and hosing with Sleeve Indexed Bayonet, can be used, or the machines can be retrofitted with a yoke. When purchasing new anaesthetic machines, models with a yoke for N_2_O cylinders should be sought if N_2_O is expected to be required;
- Appropriate storage, access, and monitoring of use of portable N_2_O cylinders, in compliance with the applicable standards and guidelines;
- Practicalities of transporting N_2_O cylinders to the point of clinical administration;
- Advantages to patients, who may be able to move around the room more freely when administered N_2_O via a cylinder;
- Development of a new protocol for portable cylinder ordering.

Last, healthcare facilities are encouraged to explore avenues to minimise residual N_2_O amounts in cylinders returned to suppliers, and to advocate to suppliers for solutions to address and avoid greenhouse gas emissions from venting—the practice of releasing unused N_2_O when cylinders are returned for refill.

### Recent progress

Since publication of the Guide in 2024, a number of Australian hospitals have decommissioned their piped N_2_O infrastructure and moved to portable supply of N_2_O.^24^ Hospitals with large birthing suites and paediatric hospitals face additional challenges in moving to portable N_2_O supply. While examples of major obstetric hospitals successfully decommissioning are awaited, The Children’s Hospital at Westmead moved to cylinder based supply of N_2_O in December 2025.

**Table:** 

**Case study: The Prince Charles Hospital, Brisbane^25,26^**
The Prince Charles Hospital, a major cardiothoracic hospital with no birthing facilities in Brisbane, decommissioned their N_2_O piped infrastructure in January 2024. By moving from piped N_2_O to portable cylinders at the point of care, hospital purchasing of N_2_O decreased by 93%, realising immediate environmental and cost savings with a return on investment of just over two years.
In 2019–2020, The Prince Charles Hospital procured a total of 584,000 l of N_2_O via manifold and point-of-care cylinders compared with 19,200 l in point-of-care cylinders only for 2023–2024. The emissions of 584,000 l and 19,200 l of N_2_O are approximately 300 and 10 tonnes (t) of CO_2_e respectively. Therefore, decommissioning the reticulated N_2_O and moving to point-of-care cylinders prevented the emission of 290 t CO_2_e per annum.
**Case study: The Children’s Hospital at Westmead, Sydney Children’s Hospital’s Network, Sydney**
The Children’s Hospital at Westmead is the largest children’s hospital in New South Wales and provides tertiary–quaternary care across all specialties. Over a two-year process, supported by staff and the executive, it measured nitrous oxide leakage across the hospital, and audited all clinical nitrous oxide usage simultaneously. Based on these data, and using an extensive consultation and education process, on 1 December 2025 it ceased using its existing piped nitrous infrastructure and transitioned to full cylinder based supply across the entire hospital, including operating theatres, emergency departments and outpatients and ward-based sedations. Full decommissioning of the pipeline infrastructure is underway as part of the Sydney Children’s Hospitals Network commitment to network wide decommissioning.
**Case study: Broome Hospital, Western Australia^27^**
Broome Hospital is a regional hospital which provides maternity services for over 300 births per year. In 2025, with strong support from their medical, midwifery and nursing staff, Broome Hospital decommissioned their piped N_2_O infrastructure and transitioned to portable cylinders in the birth suites and theatres. Broome Hospital staff note that mobile N_2_O delivery units may enhance the birthing experience by enabling greater mobility during labour.

## Conclusion

Evidence suggests that the current Australian Standards for maintenance of N_2_O infrastructure and pipelines in healthcare facilities are inadequate to detect leaks, and that as a result millions of litres of N_2_O, and unnecessary greenhouse gas emissions, are currently leaking from healthcare facility infrastructure before clinical administration. Detailed guidance was developed and is presented in the Guide to detect and reduce leaks from N_2_O in healthcare facilities, alongside case studies of hospitals from across Australia who have successfully applied these methodologies and implemented improvements. Acting to reduce N_2_O infrastructure leaks represents an important opportunity for healthcare facilities to substantially reduce their greenhouse gas emissions and associated financial costs with no impact on patient care—and a significant opportunity for the health system to show leadership in responding to the threat of climate change.
